# High-power short-duration versus standard-power standard-duration settings for repeat atrial fibrillation ablation

**DOI:** 10.1007/s00380-021-01987-9

**Published:** 2021-11-30

**Authors:** Joey Junarta, Sean J. Dikdan, Naman Upadhyay, Sairamya Bodempudi, Michael Y. Shvili, Daniel R. Frisch

**Affiliations:** 1 Department of Medicine, Jefferson Heart Institute, Thomas Jefferson University Hospital, 925 Chestnut Street, Mezzanine level, Philadelphia, PA 19107 USA; 2 Sidney Kimmel Medical College, Thomas Jefferson University, Philadelphia, USA

**Keywords:** Catheter ablation, Atrial fibrillation, Recurrent atrial fibrillation, Electrophysiology

## Abstract

**Introduction:**

High-power short-duration (HPSD) ablation is a novel strategy using contact force-sensing catheters optimized for radiofrequency ablation for atrial fibrillation (AF). No study has directly compared HPSD versus standard-power standard-duration (SPSD) contact force-sensing settings in patients presenting for repeat ablation with AF recurrence after initial ablation.

**Methods:**

We studied consecutive cases of patients with AF undergoing repeat ablation with SPSD or HPSD settings after their initial pulmonary vein isolation (PVI) with temperature controlled non-contact force, SPSD or HPSD settings between 6/23/14 and 3/4/20. Procedural data collected included radiofrequency ablation delivery time (RADT). Clinical data collected include sinus rhythm maintenance post-procedure.

**Results:**

A total of 61 patients underwent repeat ablation (36 SPSD, 25 HPSD). A total of 51 patients (83.6%) were found to have pulmonary vein reconnections necessitating repeat isolation, 10 patients (16.4%) had durable PVI and ablation targeted non-PV sources. RADT was shorter when comparing repeat ablation using HPSD compared to SPSD (22 vs 35 min; *p* = 0.01). There was no difference in sinus rhythm maintenance by Kaplan–Meier survival analysis (log rank test *p* = 0.87), after 3 or 12-months between groups overall, and when stratified by AF type, left atrial volume index, CHA_2_DS_2_-VASc score, or left ventricular ejection fraction.

**Conclusion:**

We demonstrated that repeat AF ablation with HPSD reduced procedure times with similar sinus rhythm maintenance compared to SPSD in those presenting for repeat ablation.

**Supplementary Information:**

The online version contains supplementary material available at 10.1007/s00380-021-01987-9.

## Introduction

The foundation of radiofrequency (RF) catheter ablation for atrial fibrillation lies in the electrical isolation of the pulmonary veins (PVI) from the left atrium [1, 2]. This technique is proven to be effective and safe in reducing AF burden [1, 2]. Despite durable PVI after ablation, AF can recur. Repeat studies often demonstrate PV reconnections. However, at times, AF recurs even with durable PVI. The mechanism behind PV reconnection is incompletely understood, though incomplete ablation with partial thickness lesions or reversible injury is thought to be an important contributor [3–5].

Recently, there has been increasing interest in the use of contact force-guided (CF) high-power (45 to 70 W) short-duration (5 to 15 s) (HPSD) ablation for AF to reduce its recurrence and shorten ablation times. With standard-power (20 to 40 W) standard-duration (20 to 60 s) (SPSD) settings, the incidence of AF recurrence rate is about 15% at 3 months [6, 7]. Increasing the power to 45 to 50 W has been shown to improve ablation outcomes, but increasing power without shortening delivery duration has been associated with increased complications [8]. As such, other reports have used 50 W lesions for shorter durations, which led to improved ablation outcomes without increasing complications [9].

Three recent meta-analyses of observational studies comparing HPSD versus SPSD for initial AF ablation have yielded conflicting results [10–12]. There have been only two randomized controlled trials (RCT) comparing HPSD and SPSD settings thus far, which have also yielded inconsistent results with regards to sinus rhythm maintenance [13, 14]. Whether HPSD allows for improved freedom from AF after initial ablation is still unclear.

To our knowledge, there are no studies directly comparing HPSD versus SPSD settings in patients presenting for repeat ablation with AF recurrence after initial ablation. Previously, we compared clinical and procedural outcomes between temperature-controlled non-contact force (TCNC), SPSD, and HPSD settings at our institution [15, 16]. In this report, we compared the long term clinical and procedural outcomes between HPSD and SPSD for repeat ablation in those with AF recurrence after initial AF ablation.

## Methods

### Study population and design

This consecutive case series included patients with paroxysmal or persistent AF referred for repeat ablation of AF between June 23, 2014 and March 4, 2020. Patients were eligible if they were undergoing their first repeat RF ablation with a strategy of SPSD or HPSD after undergoing an initial AF ablation with TCNC, SPSD, or HPSD settings. Patients were excluded if they underwent ablation for any other arrhythmia, if they presented for repeat ablation for AF beyond their first one, or if an ablation modality other than RF was used. Data on procedural and clinical characteristics were collected from our institution’s electronic health record and stored in a secure password-protected database. The study was approved by our institutional review board.

### Catheter ablation procedure

Written, informed consent was provided by all patients before the procedure in accordance with institutional policy. Antiarrhythmic drugs except amiodarone were stopped 3 days prior to the procedure. We have previously described our ablation protocol in separate studies [15, 16]. Briefly, femoral venous access was obtained, then a multipolar catheter was placed in the coronary sinus. Afterwards, we place a diagnostic intra-cardiac ultrasound catheter (5.5–10 MHz, AcuNav, Biosense Webster, Diamond Bar, CA or ViewFlex™, Abbott Medical, St. Paul, MN) in the right atrium. Two inter-atrial trans-septal punctures were performed, then, an ablation catheter as well as a circular mapping catheter (Spiral, St. Jude Medical, St. Paul, MN) were advanced into the left atrium. Three-dimensional electroanatomic mapping was performed using the St. Jude EnSite™ Velocity™ system (LSI, St. Jude Medical, St. Paul, MN), which is capable of recording the LSI during ablation.

Pulmonary veins were routinely isolated as a pair for initial ablation at our institution. Initial ablation was performed in the carina between ipsilateral veins if isolation could not be achieved with wide area encirclement. During repeat ablation, the pulmonary veins were first mapped to determine durability of electric isolation. Targeted PVI was conducted if reconnection occurred. In the case that pulmonary veins were durably isolated with initial mapping, the ablation strategy utilized depended on electroanatomic mapping and burst pacing inducibility for AF. Ablation targets included the anterior left atrial wall, posterior left atrial wall, left atrial roof, anterior mitral isthmus, posterior mitral isthmus, interatrial septum, cavo tricuspid isthmus, and/or coronary sinus. Generally, anterior ablation was performed if there was evidence of a re-entrant circuit or focal tachycardia originating from the anterior left atrial wall. For posterior wall ablation, either a circuit was identified on the posterior wall or high frequency, low amplitude signals were identified and targeted. Typically, the intention was complete posterior wall isolation when the posterior wall was targeted.

RF ablation for SPSD and HPSD was delivered with a 3.5-mm open-irrigated CF sensing catheter (TactiCath, St. Jude Medical, St. Paul, MN). Our SPSD protocol involved ablating with a flow of 17 cc/minute for 30–60 s, with a power of 20–25 W, at a goal of 10–40 g per lesion, and a goal of 400–500 g seconds per site, with a LSI of 4.5–5.5. Our HPSD protocol involved administering RF ablation with a flow of 30 cc/minute for up to 15 s, with a power of 50 W, at a goal of 8–40 g per lesion, guided by a LSI of 6 on the anterior left atrium and an LSI of 5 on the posterior left atrium. In all cases, esophageal temperature monitoring was arranged and lesions were aborted if the temperature rose by 0.2 °C or more.

Successful PVI was defined by the loss of all PV potentials (entrance block) and failure to capture the left atrium when pacing from sequential bipoles of the circular mapping catheter placed at the ostium of each PV (exit block; 10 millivolts were delivered with a 2 ms pulse width with each pacing stimulus). Attempts at reinduction with burst pacing were performed.

### Follow up

Patients were routinely followed-up at 1, 3, 6, and 12 months after their repeat ablation to assess for clinical outcomes. To determine AF status, patients’ reports of symptoms and electrocardiography were evaluated at each follow-up visit. We ensured conducting a high quality 12-lead electrocardiographic reading to inform further management of the patient’s AF at each visit. Mobile cardiac outpatient telemetry monitors were utilized if patients had signs or symptoms concerning for recurrence of their AF, including if they were intermittently symptomatic with chest pain, shortness of breath, palpitations, near syncope, or dizziness. Patients were also encouraged to report symptoms via telephone, email, or electronic medical record messaging.

### Study endpoints

Primary procedural endpoints include RF ablation delivery time (RADT) and the inducibility of arrhythmias after ablation. RADT is the total time that RF ablation was delivered and not the time in between lesions. Primary clinical endpoints included the recurrence of AF in the first 3 months and 12 months after ablation as well as the probability of AF recurrence over 12 months by Kaplan–Meier survival analysis. Recurrence of AF was defined as ≥ 30 s of asymptomatic or symptomatic AF.

### Statistical analyses

Means of continuous variables were analyzed with the student’s *t* test. The non-parametric Wilcoxon-Mann Whitney test was used to compare the median of variables. Categorical variables were analyzed using a Chi-squared test. The paired *t* test was used to compare the use of medications within the same group over the follow-up period. Kaplan–Meier curves and the log-rank test was used to compare atrial fibrillation recurrence. A two-sided *p* value of < 0.05 was used to determine statistical significance. Analyses were performed using STATA/SE 16.1 (College Station, TX, USA).

## Results

### Baseline characteristics

Clinical and baseline characteristics of included patients are shown in Table 1. There was no difference in age, sex, type of AF, CHA_2_DS_2_-VASc score, anti-arrhythmic drug use at different follow-up time periods, left atrial volume index (LAVI), or left ventricular ejection fraction (LVEF) between groups. There was a difference in anticoagulation use at study recruitment between HPSD and SPSD groups (100.0% vs. 80.0%; *p* = 0.01). However, there was no difference in anticoagulation use at 3 (*p* = 0.07) and 12-month (*p* = 0.18) follow-up.

**Table 1:** Clinical characteristics

	Standard-power standard-duration (*N* = 36)	High-power short-duration (*N* = 25)	*p* value
Age in years, mean (SD)	61.0 (1.7)	63.4 (1.7)	0.35
Male sex, no. (%)	22 (61.1%)	13 (52.0%)	0.48
Paroxysmal atrial fibrillation, no. (%)	13 (36.1%)	14 (56.0%)	0.16
CHA_2_DS_2_-VASc score, median (IQR)	2 (1–3)	1.5 (1–3)	0.85
Antiarrhythmic drug use, no. (%)	31 (86.1%)	20 (80.0%)	0.53
Antiarrhythmic drug use at 3 months follow-up, no. (%)	27 (75.0%)	20 (80.0%)	0.44
Antiarrhythmic drug use at 12 months follow-up, no. (%)	22 (61.1%)	17 (68.0%)	0.38
Anticoagulant use, no. (%)	36 (100.0%)	20 (80.0%)	0.01
Anticoagulant use at 3 months follow-up, no. (%)	31 (86.1%)	16 (64.0%)	0.07
Anticoagulant use at 12 months follow-up, no. (%)	27 (75.0%)	14 (56.0%)	0.18
Left atrial volume index, mean (SD)	36.9 (2.4)	35.7 (2.6)	0.76
Left ventricular ejection fraction %, mean (SD)	58.8 (12.7)	61.0 (12.2)	0.51

*N* number of participants, *SD* standard deviation, *no.* number, *IQR* inter-quartile range

### Procedural outcomes

A total of 24 patients (39.3%) underwent their initial RF ablation with TCNC. A total of 34 (55.7%) and 3 (4.9%) patients underwent initial RF ablation with SPSD and HPSD settings, respectively. Upon evaluation during repeat ablation, 51 patients (83.6%) were found to have pulmonary vein reconnections necessitating repeat isolation, while 10 patients (16.4%) had durable PVI and ablation targeted non-PV sources (Supplementary Table 1). Non-PV sources that were targeted included ablation of the anterior and posterior mitral isthmus, interatrial septum, coronary sinus, left atrial roof line, anterior left atrial wall, posterior left atrial wall, and cavo tricuspid isthmus.

**Fig. 1:**
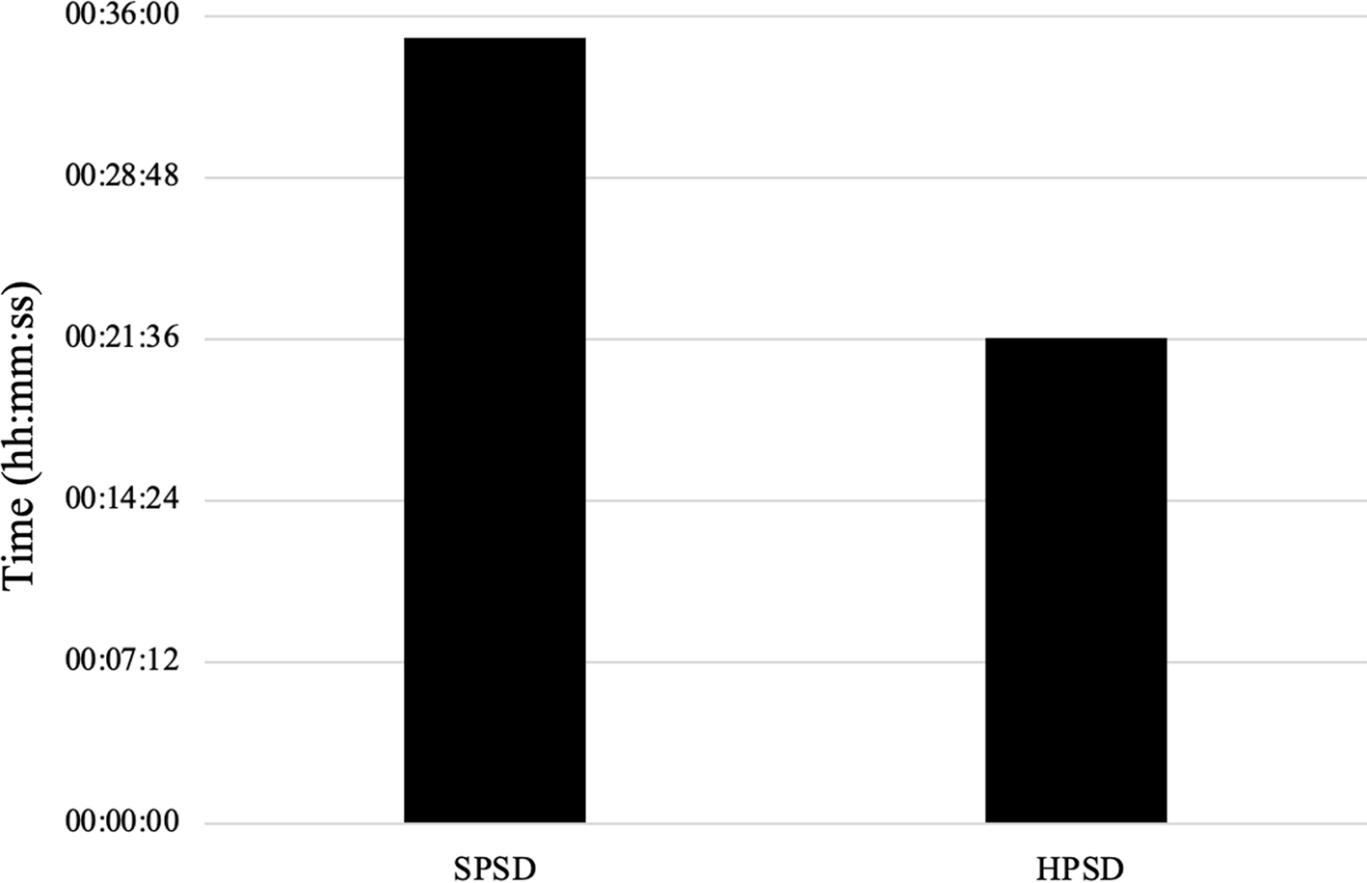
Radiofrequency ablation delivery time by ablation settings. *SPSD* standard-power standard-duration, *HPSD* high-power short-duration

Table 2 and Fig. 1 compares procedural outcomes between catheter settings. There was a difference in RADT between groups (*p* = 0.01). HPSD settings reduced ablation times by about 13 min. There was no difference in the ability to reinduce arrhythmias between catheter types (Table 3).

**Table 2:** Procedural time by catheter type

	Radiofrequency ablation delivery time	
	Standard-power standard-duration	High-power short-duration
N	36	25
Mean	00:35:03	00:21:38
SD	00:21:56	00:15:12
p value	0.01	

*N* number of participants, *SD* standard deviation

**Table 3:** Arrhythmia inducibility by ablation strategy

	Standard-power standard-duration (*N* = 36)	High-power short-duration (*N* = 25)	*p* value
Non-inducible	20	14	0.32
Atrial fibrillation	8	9	
Other supraventricular arrhythmia	7	2	

*N* number of participants

### Clinical outcomes

**Fig. 2:**
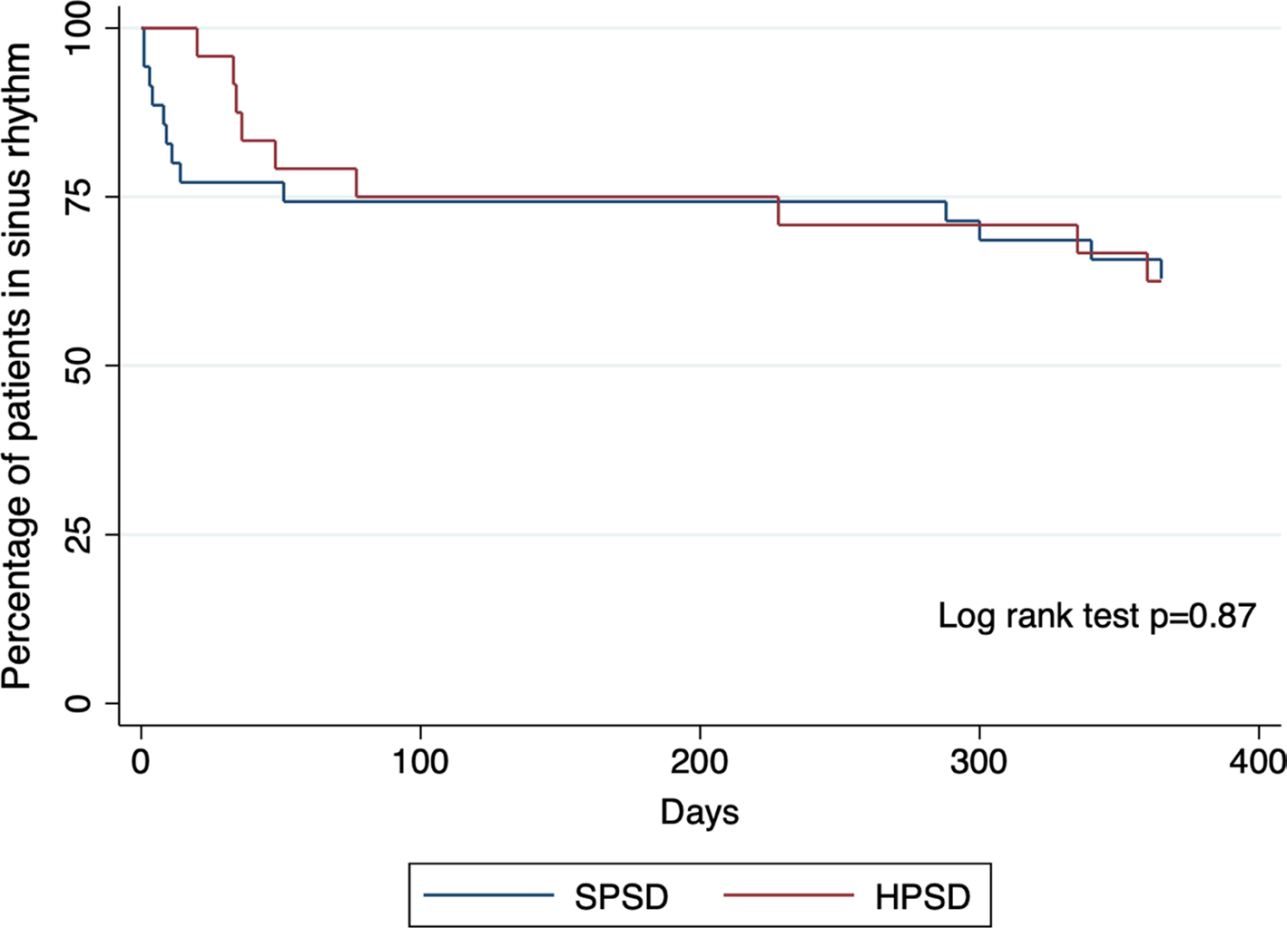
Kaplan–Meier survival analysis for atrial fibrillation recurrence. *SPSD* standard-power standard-duration, *HPSD* high-power short-duration

AF recurrence was assessed at 3 months and 12 months after ablation. There was no difference in the overall percentage of patients in sinus rhythm between groups at 3 months (Table 4) or 12 months (Table 5). Notably, the number of patients in both groups decreased over time as they were lost to follow-up. There was no difference in AF recurrence when patients were stratified by AF type, LAVI, CHA_2_DS_2_-VASc score, or LVEF at 3 months or 12 months. Time to first AF recurrence for each patient was assessed and showed no difference between groups over 12 months by Kaplan–Meier survival analysis (log rank test *p* = 0.87) shown in Fig. 2.

**Table 4:** Patients in sinus rhythm after 3 months based on clinical characteristics and catheter type

	SPSD (*N* = 35)	HPSD (*N* = 24)	*p* value
Overall patients in sinus rhythm, no. (%)	27 (77.1%)	19 (79.2%)	0.85
Type of atrial fibrillation, no			
Paroxysmal	10	11	0.76
Persistent	18	9	
Left atrial volume index, no			
≥ 35	10	7	1.00
< 34	10	7	
CHA_2_DS_2_-VASc score, no			
≥ 2	17	12	0.84
< 2	10	8	
Left ventricular ejection fraction %, no			
≥ 55	17	17	0.13
< 55	9	3	

*SPSD* standard-power standard-duration, *HPSD* high-power short-duration, *N* number of participants, *no.* number

**Table 5:** Patients in sinus rhythm after 12 months based on clinical characteristics and catheter type

	SPSD (*N* = 33)	HPSD (*N* = 24)	*p* value
Overall patients in sinus rhythm, no. (%)	23 (69.7%)	15 (62.5%)	0.57
Type of atrial fibrillation, no			
Paroxysmal	10	10	0.64
Persistent	13	5	
Left atrial volume index, no			
≥ 35	11	4	0.26
< 34	8	7	
CHA_2_DS_2_-VASc score, no			
≥ 2	17	9	0.45
< 2	6	6	
Left ventricular ejection fraction %, no			
≥ 55	17	13	0.46
< 55	6	2	

*SPSD* standard-power standard-duration, *HPSD* high-power short-duration, *N* number of participants, *no.* number

### Adverse events

No adverse events occurred. No pericardial effusion, esophageal injuries, phrenic nerve injuries, bleeding requiring transfusion, strokes, or deaths occurred in any groups.

## Discussion

Our study demonstrates that HPSD reduced procedural times compared to SPSD ablation with no difference in sinus rhythm maintenance in patients presenting for repeat RF ablation for AF. Additionally, there was no difference in sinus rhythm maintenance when stratified by AF type, left atrial volume index, CHA_2_DS_2_-VASc score, or left ventricular ejection fraction.

There are two heating phases that constitute the basis of RF ablation lesion formation: resistive and conductive heating. The electrical current delivered during resistive heating leads to immediate and irreversible injury to superficial tissue layers at temperatures of > 50 °C. During the resistive heating phase, a heat source created passively extends into deeper tissue layers at lower temperatures of < 50 °C, this is known as conductive heating. It has been shown that high-power RF (45 to 70 W) delivery with shorter duration (5 to 15 s) may lead to a shift allowing increased resistive heating and decreased conductive heating, and be sufficiently adequate in creating effective ablation lesions [17, 18]. The superiority and safety of HPSD is of considerable interest.

Three meta-analyses of observational studies and two RCTs comparing HPSD versus SPSD for initial AF ablation have yielded conflicting results [10–14]. The meta-analyses included anywhere between 10 and 15 studies involving 2274 to 3718 patients [10–12]. Two showed a difference in freedom from atrial arrhythmia with HPSD when compared to conventional RF settings [10, 11]. However, the meta-analysis by Kewcharoen et al. did not [12]. Nevertheless, these studies were consistent in demonstrating reduced RF ablation time with HPSD settings and showed no difference in peri-procedural complications.

Less studied are the optimal ablation parameters that should be used for ablation of recurrent AF. In one single cohort study, the outcome of repeat RF ablation with 35 W of energy after initial cryoballoon ablation was evaluated [19]. By means of Kaplan–Meier analysis, freedom from AF was 94% and 86% at 12 months and 24 months after redo ablation, respectively. Pokushalov et al. compared cryoballoon versus RF ablation of paroxysmal AF after failed initial RF ablation [20]. Repeat AF ablation was done with 35 W of energy. By intention-to-treat analysis, 58% of the RF group vs 43% of the cryoballoon group (*p* = 0.06) patients were AF-free on no antiarrhythmics. With regards to on-treatment comparisons, 53% of patients in the RF group and 38% of the cryoballoon group (*p* = 0.04) were AF-free at 12-month follow-up. This study suggests that RF may be preferable to cryoballoon as a redo ablation strategy.

No studies have directly compared HPSD versus SPSD settings in patients presenting for repeat ablation with AF recurrence after initial RF ablation. We demonstrate that HPSD shortened ablation times without sacrificing efficacy. Perhaps not surprisingly, our study did not show improved clinical outcomes using HPSD in patients presenting for redo ablation. It is important to emphasize that although suggestive, there is no convincing randomized evidence that HPSD ablation allows for improved long-term outcomes, be it for initial or redo ablations.

A further observation is the recurrence pattern based on the strategy used for the initial ablation. Despite the development of CF measurements, automated lesion assessment, and next-generation catheters, AF recurrence after PVI remains a significant challenge. Here, we showed that most patients presenting for redo ablation had evidence of PV reconnection (83.6%). Herein lies the interest in optimizing RF settings for improved outcomes after RF ablation.

### Limitations

Our study had a small sample size, which meant that it was difficult to detect a significant difference in clinical outcomes between groups. Next, it is possible that some patients had undetected AF recurrence, which could falsely elevate the rate of sinus rhythm maintenance in the study. This is despite close follow-up, outpatient electrocardiographic monitoring, and telemetry monitoring, as rhythm monitoring was not continuous. There was a baseline difference in anticoagulant use between groups. Importantly, there was no difference in the use of anticoagulant drugs at 3 and 12 months between groups, which minimizes any potential impact this may have on confounding the long-term maintenance of sinus rhythm attributed to the different ablation strategies.

## Conclusion

To the best of our knowledge, this is the first study to compare HPSD versus SPSD settings in patients presenting for repeat AF ablation after AF recurrence. We found that AF ablation with HPSD settings guided by LSI reduced procedure times with similar sinus rhythm maintenance and safety profile compared to SPSD for those undergoing their first redo ablation. Further large RCTs will help clarify whether HPSD settings improve not only procedural times, but long term sinus rhythm maintenance in those presenting for initial and redo RF ablation for AF.

## Supplementary Information

Below is the link to the electronic supplementary material.

Supplementary file1 (DOCX 13 KB)

## Data Availability

Data are safely kept in a password protected security system at Thomas Jefferson University Hospital. The datasets used and/or analysed during the current study are de-identified and available from the corresponding author on reasonable request.
