# Supplementary material for: High-power short-duration versus standard-power standard-duration settings for repeat atrial fibrillation ablation
Source: Heart Vessels. 2021 Nov 30;37(6):1003–9. doi: 10.1007/s00380-021-01987-9 (PMC9114024; doi:10.1007/s00380-021-01987-9)
Supplement: Supplementary file 1 — Supplementary file1 (DOCX 13 KB) [file 380_2021_1987_MOESM1_ESM.docx]

**Supplemental Table 1.** Non-pulmonary vein ablation targets.

| Patient | Ablation target |
| --- | --- |
| 1 | Anterior and posterior mitral isthmus, interatrial septum, coronary sinus, left atrial roof line, posterior left atrial wall |
| 2 | Anterior and posterior mitral isthmus |
| 3 | Anterior and posterior mitral isthmus, posterior left atrial wall, cavo tricuspid isthmus |
| 4 | Anterior and posterior left atrial wall |
| 5 | Posterior left atrial wall |
| 6 | Posterior left atrial wall |
| 7 | Left atrial roof line |
| 8 | Anterior and posterior mitral isthmus |
| 9 | Cavo tricuspid isthmus |
| 10 | Cavo tricuspid isthmus |
